# Supplementary material for: Graph neural network-based anomaly detection for river network systems
Source: F1000Res. 2024 Mar 28;12:991. Originally published 2023 Aug 16. [Version 2] doi: 10.12688/f1000research.136097.2 (PMC11162521; doi:10.12688/f1000research.136097.2)
Supplement: Peer review history

# Reviewer report: Reviewer response for version 2

**DOI:** 10.5256/f1000research.162799.r282583

**Published:** 2024-06-08

**Authors:** J Vellingiri

**Affiliations:** 1 Vellore Institute of Technology, Vellore, Tamil Nadu, India

The author focuses on the importance of anomaly detection to predict water quality. Anomaly detection is critical to detect false patterns in sensor data. And it highlights a difficult process because of the data's complexity and variability. Author proposed a GDN-based method. Overall work is good, I have **accepted the proposed work with minor revisions**.

**Minor points:**

 1. Author only discussed GDN methods. Might include related work parts and discuss other relevant algorithms.

 2. Problem statement part needs to be added precisely.

 3. In the method section the author might clarify why Threshold-based Anomaly detection is used.

Is the work clearly and accurately presented and does it cite the current literature?

Yes

If applicable, is the statistical analysis and its interpretation appropriate?

I cannot comment. A qualified statistician is required.

Are all the source data underlying the results available to ensure full reproducibility?

Yes

Is the study design appropriate and is the work technically sound?

Yes

Are the conclusions drawn adequately supported by the results?

Yes

Are sufficient details of methods and analysis provided to allow replication by others?

No

Reviewer Expertise:

Data Analytics

I confirm that I have read this submission and believe that I have an appropriate level of expertise to confirm that it is of an acceptable scientific standard.

# Reviewer report: Reviewer response for version 1

**DOI:** 10.5256/f1000research.149233.r208772

**Published:** 2023-10-20

**Authors:** Maurício Araújo Dias

**Affiliations:** 1 Department of Mathematics and Computer Science, School of Sciences and Technology, Universidade Estadual Paulista, São Paulo, State of São Paulo, Brazil

1. In recent years, there has been an increasing interest in distinguishing anomaly detection from outlier detection (Chandola *et al.*, 2009 ^1^; Chandola *et al.*, 2012 ^2^; Kittler *et al.*, 2014 ^3^; Dias *et al.*, 2019 ^4^; Dias *et al.*, 2022 ^5^). According to the scientific literature, the former differs from the latter by categorizing outliers based on some taxonomy, in addition to just detecting them. In 2009 and 2010, a small taxonomy was used in Chandola *et al. *(2009 ^1^) and Chandola *et al. *(2012 ^2^). In 2014, Kittler’s Taxonomy, a more complete taxonomy, was introduced in Kittler *et al.* (2014 ^3^). In 2020, Kittler’s Taxonomy was used as a basis to detect anomalies (high variability) mainly in rivers and in a spatial context, as described in Dias *et al.* (2019 ^4^). In 2022, a detection of anomalies (drift) based on Kittler’s Taxonomy, in a river and mainly in a temporal context, was described in Dias *et al.* (2022 ^5^).
  The reviewed article, which presents a graph neural network-based anomaly detection for river network systems, is in agreement with the aforementioned papers. In the reviewed article, the anomaly detection process deals with the complexity and variability of the data from spatio-temporal relationships between sensors for accurate and continuous monitoring of river water quality. Another positive aspect of the reviewed article is that it actually describes anomaly detection, in contrast to many articles present in the scientific literature that describe outlier detection despite using the term anomaly detection. Moreover, the work is clearly and accurately presented. A negative aspect of the reviewed article is that it could have had more references on this distinguishing and on anomaly taxonomies. It would also be interesting to highlight in the beginning of the article which taxonomy the authors based on to categorize anomalies as being of the following types: high-variability, drift, spikes, and shifts (ref. 7 cited by the article maybe?).
2. In general, the study design is appropriate. My only suggestions are, please: (1) To add north arrow and scale bar to Figures 1, 2, and 7. (2) To consider presenting the algorithm in page 9 (Algorithm 2, maybe?) in the same style that Algorithm 1 was presented in page 8. The work is technically sound.
3. There are sufficient details of methods and analysis provided to allow replication by others. The reviewed article provides researchers with many mathematical definitions regarding the steps of the methodology used in the work. Those mathematical definitions allow researchers to replicate the work more accurately. The analysis process is supported by a number of real and simulated benchmark data.
4. The statistical analysis and its interpretation is appropriate since the work validated its results based on well-established metrics, as follows: (1) The quantity of truly relevant results was measured using Recall. (2) The efficiency of results was measured using Accuracy. (3) The relevancy of results was measured using Precision. (4) The ratio of true negatives to all negative outcomes was measured using Specificity.
5. The source data and software underlying the results of this work are all available on the Internet to ensure full reproducibility. The authors add “Data Availability” and “Software Availability” subsections to the end of the paper. My only suggestion is to distinguish and highlight “real” and “simulated” benchmark data in the subsection “Data Availability”.
6. The conclusions drawn are adequately supported by the results. However, I would like to highlight a certain feature present in the reviewed article, although I do not particularly consider it a flaw. The contents of the “Discussion” and “Conclusions” sections of the reviewed article are most commonly found in the scientific literature merged to form a broader “Conclusions” section than that presented in the reviewed article. For example, suggestions for future work are normally part of the “Conclusions”, but in the reviewed article they appear in the “Discussion” (in a scientific article, it seems a bit strange to “discuss” something that has not yet been done). The scientific literature usually discusses the results found. This is done in the reviewed article, but merged with the “Results” section, rather than in the “Discussion”.

Is the work clearly and accurately presented and does it cite the current literature?

Partly

If applicable, is the statistical analysis and its interpretation appropriate?

Yes

Are all the source data underlying the results available to ensure full reproducibility?

Yes

Is the study design appropriate and is the work technically sound?

Yes

Are the conclusions drawn adequately supported by the results?

Yes

Are sufficient details of methods and analysis provided to allow replication by others?

Yes

Reviewer Expertise:

NA

I confirm that I have read this submission and believe that I have an appropriate level of expertise to confirm that it is of an acceptable scientific standard, however I have significant reservations, as outlined above.

## References

[1] : Anomaly detection. *ACM Computing Surveys* .2009;41(3) : 10.1145/1541880.1541882 1-58 10.1145/1541880.1541882

[2] : Anomaly Detection for Discrete Sequences: A Survey. *IEEE Transactions on Knowledge and Data Engineering* .2012;24(5) : 10.1109/TKDE.2010.235 823-839 10.1109/TKDE.2010.235

[3] : Domain Anomaly Detection in Machine Perception: A System Architecture and Taxonomy. *IEEE Trans Pattern Anal Mach Intell* .2014;36(5) : 10.1109/TPAMI.2013.209 845-59 10.1109/TPAMI.2013.209 26353221

[4] : An Incongruence-Based Anomaly Detection Strategy for Analyzing Water Pollution in Images from Remote Sensing. *Remote Sensing* .2019;12(1) : 10.3390/rs12010043 10.3390/rs12010043

[5] : A Machine Learning Strategy Based on Kittler’s Taxonomy to Detect Anomalies and Recognize Contexts Applied to Monitor Water Bodies in Environments. *Remote Sensing* .2022;14(9) : 10.3390/rs14092222 10.3390/rs14092222

# Response

**Published:** 2024-01-26

**Authors:** Katie Buchhorn

**Affiliations:** School of Mathematical Science, Queensland University of Technology, Brisbane, Queensland, Australia

The authors thank the reviewer for their thoughtful feedback. Please see the enumerated responses below:

 1. Clarification of the anomaly taxonomy has been added in the introduction, including reference 7. Thank you for highlighting these papers. We considered the inclusion of the above references, however since as their definition of “drift” differs, they were not included to avoid confusion.

 2. North arrow and scale have been added to Figure 7 (Figure 1 & 2 are simulated data with arbitrary values for the scale and direction). Algorithm 1 is pseudo-code, whereas on the following page, an excerpt of python code is provided. Thus, we feel it is necessary to have a distinction in presentation.

 5. The “Data availability” section has been updated with this clarification.

# Reviewer report: Reviewer response for version 1

**DOI:** 10.5256/f1000research.149233.r199872

**Published:** 2023-09-28

**Authors:** Ailin Deng

**Affiliations:** 1 National University of Singapore, Singapore, Singapore

The authors provide detailed analysis on a previous anomaly detection method, GDN and improve it by proposing a new threshold selection methodology based on the learned local graph structure, denoted as GDN+. The work mainly focuses on the river network systems and further provides benchmarking data generation which is specifically designed for the river network or similar systems' analysis. They show the GNN-based method’s effectiveness in the river network systems in their study.

 Pros:

- The threshold selection based on the neighborhood sensors is interesting and reasonable. It enables a more flexible threshold for sensors based on the learned graph structure.
- The authors provide detailed code implementation and python package for the method.
- They also provide methods for generating data based on spatio-temporal structures.
- The case study is interesting and effective to show the interpretability in this river system scenario.

Cons:

- It would be interesting to have an ablation study on the effect of the \tau selection, it seems the default setting is 99 (or should it be 99%?), it would be good to see how different \tau will affect the recall or other performance metrics.

Minor:

- In table 2, some of the methods are bold, but it seems they are not achieving the best recall performance, such as ARIMA.
- In GDN+ threshold part, should the denotation exclude i for A_{ji}? As the A_{ij} includes {i=j} and the description is “across the neighborhood of i” which might not include sensor i itself.
- Some of the claims could be rephrased more gently or targeted at a more specific domain of problem, such as anomaly detection in river network systems. Page 4 of 18: “Lack of open-source software for anomaly detection…”. For anomaly detection, there are some existing open-source packages, such as PyOD (Zhao *et al.*, 2019 ^1^).

In overall, I think this paper has a clear writing and detailed study in anomaly detection in the specific river system and provides open-source code for method and data generation for this kind of problem. Some ablation study and minor or typos can be further addressed.

Is the work clearly and accurately presented and does it cite the current literature?

Partly

If applicable, is the statistical analysis and its interpretation appropriate?

Not applicable

Are all the source data underlying the results available to ensure full reproducibility?

Yes

Is the study design appropriate and is the work technically sound?

Yes

Are the conclusions drawn adequately supported by the results?

Partly

Are sufficient details of methods and analysis provided to allow replication by others?

Yes

Reviewer Expertise:

Anomaly detection

I confirm that I have read this submission and believe that I have an appropriate level of expertise to confirm that it is of an acceptable scientific standard, however I have significant reservations, as outlined above.

## References

[1] : PyOD: A Python Toolbox for Scalable Outlier Detection. *JMLR* .2019;20(96) :1-7 Reference source

# Response

**Published:** 2024-01-26

**Authors:** Katie Buchhorn

**Affiliations:** School of Mathematical Science, Queensland University of Technology, Brisbane, Queensland, Australia

The authors thank the reviewer for their thoughtful feedback. Please see the responses below:

 Cons:

 - Tau is introduced as a percentile (as opposed to percentage), and ranges between 0-100.  As per the suggestion, a complete ablation study has been added to the Appendix.

 Minor:

 - We utilised bold to highlight points of discussion (particularly poor or particularly good performance). However, to avoid confusion the choice of formatting has been updated so that bold denotes the highest recall value, and underline denotes the second highest.

 - Yes, the GDN+ threshold notation should exclude i for A_{ji}, this has been amended.

 - Wording has been changed to “Limited open-source software for anomaly detection…”.
